# Assessment of water consumption during Ramadan intermittent fasting: Result from Indonesian cross-sectional study

**DOI:** 10.3389/fnut.2022.922544

**Published:** 2022-07-25

**Authors:** Diana Sunardi, Dian Novita Chandra, Bernie Endyarni Medise, Dewi Friska, Nurul Ratna Mutu Manikam, Wiji Lestari, Putri Novia Choiri Insani, Amelya Augusthina Ayusari, Diana Mayasari, Fitria Saftarina, Dina Keumala Sari, Yuliana Noor Setiawati Ulvie

**Affiliations:** ^1^Department of Nutrition, Faculty of Medicine Universitas Indonesia – Dr. Cipto Mangunkusumo Hospital, Jakarta, Indonesia; ^2^Indonesian Hydration Working Group, Faculty of Medicine Universitas Indonesia, Jakarta, Indonesia; ^3^Department of Child Health, Faculty of Medicine Universitas Indonesia – Dr. Cipto Mangunkusumo Hospital, Jakarta, Indonesia; ^4^Occupational Medicine, Department of Community Medicine, Faculty of Medicine Universitas Indonesia, Jakarta, Indonesia; ^5^Faculty of Medicine, Universitas Sebelas Maret – Dr. Moewardi Hospital, Surakarta, Indonesia; ^6^Faculty of Medicine, Universitas Lampung, Bandar Lampung, Indonesia; ^7^Faculty of Medicine, Universitas Sumatera Utara, Medan, Indonesia; ^8^Department of Nutrition, Faculty of Nursing and Health Science, Universitas Muhammadiyah Semarang, Semarang, Indonesia

**Keywords:** Ramadan fasting, hydration, fluid intake, intermittent fasting, drinking pattern

## Abstract

During Ramadan fasting, people are likely to consume water and beverages lower than recommended intake due to the limited time. However, it is necessary to achieve the recommended daily water intake to maintain the hydration status, as well as productivity during fasting. Unfortunately, there is a lack of data on drinking patterns during Ramadan. This study aims to investigate water and beverage intake and drinking patterns to help achieve water requirements during Ramadan among Indonesian adults. This is a cross-sectional study conducted during the Ramadan period from April to May 2021 (Ramadan 1442 Hijri). We used a self-administered questionnaire on drinking habits during Ramadan and utilized a 7-day fluid record (Liq.In 7) to assess water and beverage intake among participants who were managed through online procedure. There were 380 participants from five universities across Indonesia who completed the questionnaire accordingly and then analyzed it. The result shows that total water and beverage intake during Ramadan among participants was below the recommendation [1,670 (1,326–2,034) ml/day]. Among the type of beverages, water is the highest level of consumption [1,262 (983–1,666) ml/day] then followed by sugar-sweetened beverages [200 (91–350) ml/day]. We found a significant difference in water and beverages consumption between time of iftar [474 (375–590) ml/day], nighttime [574 (414–810) ml/day], and suhoor [560 (423–711) ml/day]. From this study, we found that during Ramadan the most common drinking pattern is 2-4-2, but a drinking pattern of 4-2-2 glasses (sequence of four glasses at iftar, two glasses at nighttime, two glasses at suhoor) had a significantly higher chance to adhere with the recommendation of fluid intake compared to other patterns. Therefore, based on this research on water and beverage intake, it is necessary and important to make improvements among Indonesian adults during Ramadan, and the drinking pattern of 4-2-2 glasses may help to achieve the recommended daily water consumption.

## Introduction

Water is one of the important nutrients, which is frequently overlooked among others. Sufficient amount of water in the body is needed because water is an essential component for normal human body function. The loss of body water by 2% can decrease the alertness, mood, and mental state (1, 2). Dehydration leads to declining cognitive and aerobic performance (3). In addition, chronic dehydration may affect the kidneys to function over the course of time as a study showed the relationship between water intake and kidney stones, chronic kidney disease, and urinary tract infection (4, 5). Water has also been proven to be a potential protective factor from obesity, cardiovascular disease, and diabetes mellitus (6, 7).

Indonesian Liq.In7 survey in 2016 showed that water intake among 18–65-year-old adults was 2,599 ml/day (8), which is higher than the recommended intake for Indonesians, where women are recommended to consume 1,888 ml/day and men to consume 2,000 ml/day (9). Nevertheless, in that study, 28% of adults did not achieve the recommendation. Based on sex, more women achieved water recommendation compared to men (75% vs. 67%) (8). A study by Sunardi et al. on fluid intake during the pandemic among workers showed that total water intake was 1,882 (1,473–2,433) ml/day, which was lower than the survey in 2016 (10).

Ramadan fasting is observed annually by adult Muslims for 1 month. The fasting begins with pre-dawn meal (suhoor) and finishes in the evening with breakfasting (iftar). The length of the Ramadan fasting time varies based on the geographical and solar seasons (11). In Indonesia, generally the fasting duration takes approximately 13 h, from 5 a.m. to 6 p.m. (12). During daytime, between suhoor and iftar, fasting Muslims are mandated to abstain from foods and drinks, even drinking water. This condition leads to limited time for drinking water (only 11 h from iftar to suhoor) and therefore may potentially lead to low fluid intake (13). There are time restrictions for eating and drinking during Ramadan; nonetheless, Ramadan has been proven to be beneficial for health. Jahrami et al. reported that there was an improvement in lipid profile among adults who were fasting during Ramadan. The total cholesterol and triglyceride decreased, along with an increase in high-density lipoprotein (14). This result was also established in the study among overweight and obese adults where fasting during Ramadan has improved subjects’ lipid profile (15). Ramadan fasting was also shown to be beneficial for waist circumference, blood pressure, and body weight enhancement (16, 17).

A previous study among physically active men showed that four out of five studies presented a decreased intake of water during Ramadan compared to before Ramadan while one study presented no change in water intake before to during Ramadan (12). Another review by Osman et al., who investigated some studies regarding hydration status and water intake before and during Ramadan, found that the change was inconclusive due to the distinctive habits and physical activity patterns of people who are fasting during Ramadan (18). Regardless of the limited time provided during Ramadan, it is of utmost necessity to achieve the daily recommended water intake in order for the human body to function optimally during the fasting period as there is no working moderation during the Ramadan month except for reduced working hours in Indonesia. This study aimed to assess water intake among adult fasting population and their drinking pattern during Ramadan fasting. The results of this study are expected to become a recommendation for a drinking plan for intermittent fasting, especially during Ramadan.

## Materials and methods

### Design and study population

This was a cross-sectional study, where recruitment of the study population was started in the middle of April 2021 and the data collection began by the end of April 2021. All the data collection was conducted online, and data analyses were observed in Jakarta.

Participants’ eligible criteria were adults aged 18–45 years and registered as students or employees from five universities, including Universitas Sumatera Utara (USU), Universitas Lampung (UNILA), Universitas Indonesia (UI), Universitas Muhammadiyah Semarang (UNIMUS), and Universitas Sebelas Maret (UNS). The five universities were targeted because they are part of the Indonesian Hydration Working Group (IHWG), a working group that focuses on research and education program for healthy hydration. Participants were required to fill in Google Form, as data collection instruments, and in addition participants had to be able to communicate online with the enumerator, not have metabolic diseases related to liver or kidney impairment, and do Ramadan fasting during data collection.

### Procedures

Ethical clearance was provided by the Ethics Committee of the Faculty of Medicine, Universitas Indonesia–Cipto Mangunkusumo Hospital (no. 21-04-346). The whole procedure of this study was managed according to the Helsinki Declaration.

Data collection was completed by distributing information to the five universities. There was a link to access the informed consent and initial questionnaire for those who intended to participate in the study. Description, instruction, and agreement of the study were described and attached to the informed consent form. The initial questionnaire consisted of demographic characteristics, water and beverage drinking habits during Ramadan fasting, history of disease and medication, record of body weight and body height, and knowledge regarding healthy hydration. The questionnaire that was utilized in this study had been validated.

We contacted the eligible participants for further data collection. Participants recorded their fluid intake using Liq.In7 instrument for seven consecutive days in the Google Form that can be accessed by each participant (19). Data of physical activity were drawn on the fifth day of fluid intake data collection using General Physical Activity Questionnaire (GPAQ) (20). Enumerators were in charge to guide the participants on how to fill in the fluid record and GPAQ, as well as reminding them to complete their fluid record on a daily basis. Enumerators were dietitians who had been trained, respecting the form and questionnaire prior to the start of this study.

During 7-day fluid record, participants who filled the Liq.In7 properly for 4 days were considered to have completed the process. Participants who consumed water and beverages on all three occasions (iftar—nighttime—suhoor) in a day were considered to fill the Liq.In7 properly. The minimum sample size was determined by using a sample survey formula where the level of significance was 95% and power was 90% (21). Adults’ proportion who consumed water adequately was referred to Laksmi et al. Stratified sample size was conducted based on the provinces where the university is located (8). The total minimum sample size required was 361 participants.

### Measurements

The 7-day fluid record (Liq.in7) utilized in this study was adapted from Johnson (2017), where its validity had been well proven (19). In this study, we reformed it into an online record due to the pandemic situation and condition where the participants and researchers were expected to minimize direct contact with each other. Liq.in7 questionnaire included the questions of (1) time, (2) occasion, (3) beverage consumption, (4) serving size, (5) frequency of consumption, and (6) drinking location. This questionnaire was filled out after the beverages consumption and the end of the day, and the enumerators verified in which all consumptions were well recorded.

The fluid intake time was filled in based on the hour and minutes when the beverage was consumed. The answer for fluid intake occasion had three options, namely, (1) breakfasting (iftar), (2) nighttime, and (3) pre-dawn meal (suhoor). The water and beverage consumption 1 h after iftar and not during suhoor was considered a nighttime occasion. The amount of fluid consumed was obtained by multiplying the serving size and the consumption frequency, as asked in questions 4 and 5. Pictures of the containers were provided as a reference to the participants where they were able to choose one of the options or fill “other type of container” if their container was not available on the options and describe the container.

The type of beverages was an open question, so participants could freely answer about the type of beverages they had consumed. Later, the enumerator classified it into six groups, namely, (1) water, (2) hot beverages, (3) milk and derivatives, (4) sugar-sweetened beverages (SSBs), (5) juices, and (6) others. This classification was in reference to Guelinckx et al. with modification (22). The water group included mineral water, infused water, and tap water. Hot beverages consisted of tea or coffee without added sugar. Milk and derivatives consisted of whole milk, processed milk, skim milk, flavored milk, ready-to-drink milk, and yogurt; however, condensed milk was not included as it consists of high sugar content. SSBs included tea, coffee, or juices with additional sugar, ready-to-drink beverages, and condensed milk. Other beverages consisted of soy milk, herbal drink, and various Indonesian sweet sorbets.

Participants’ knowledge was measured by a questionnaire where it had been validated by Bardosono et al. (23). The questionnaire included eight questions regarding the effect of dehydration, when to drink, types of beverages, as well as drinking recommendations. Participants’ knowledge was classified as low if the score was lower than the median score of all participants (60.6 points).

Aside from the fluid record, participants were also asked in regard to their drinking pattern, which is commonly followed for water consumption. The options were the pattern of drinking in the order of iftar—nighttime—suhoor with the amounts of glasses. There were options of 2-2-2 glasses, 2-2-4 glasses, 2-4-2 glasses, 4-2-2 glasses, and no pattern. The recommendation of daily water intake is eight glasses per day, but we decided to add a pattern of 2-2-2 since during Ramadan fasting there are three eating occasions (i.e., iftar, nighttime, and suhoor), and people usually take a glass of water before eating and a glass after. Physical activity was measured by GPAQ and its validity had been studied in many countries (24). The World Health Organization (WHO) recommends the weekly active physical activity to be 600 metabolic equivalent (MET) or higher. The activity of respondents was classified as low if MET/week were lower than 600 and high if MET/week were 600 or higher (20).

Nutritional status was determined using the body mass index (BMI), which was calculated from self-reported weight and height, as Davies et al. (25) suggested that self-reported weight and height are reliable enough compared to direct observation when direct measurement is not feasible. BMI was calculated based on the formula weight (kg)/height (m^2^). The classification of BMI was referred to WHO for Western Pacific Region (2002), where the groups are (1) underweight (BMI < 18.5), (2) normal (BMI 18.5–22.9), (3) overweight–at risk (BMI 23.0–24.9), (4) overweight–obese I (BMI 25.0–29.9), and (5) overweight–obese II (BMI = 30.0). We have divided the participants into four groups where obese I and obese II groups were merged into one category, i.e., obese.

### Statistical analyses

We included the participants who completed the Liq.In7 for 4 days or more and filled out the three occasions, namely, (1) suhoor, (2) iftar, and (3) nighttime. Participants who did not complete the initial questionnaire and GPAQ were removed from the analysis. Daily fluid intake as the main outcome of this study was obtained by accumulating all fluid consumption and dividing the total consumption by the number of completed days. Total fluid consumption of more than 4,000 ml/day was excluded. Daily fluid intake is shown in ml/day.

The classification of the total water and beverage intake adequacy was coherent with the Indonesian RDA released by the 9. The total recommendations decreased by 20% because water and beverages contributed 80% of total fluid intake and the remaining 20% was from food. This study did not include water from food (8). Based on this consideration, female participants were classified into adequate water intake groups for consuming 1,880 ml/day or more and 2,000 ml/day or more for male participants.

Data normality was assessed by using Kolmogorov–Smirnoff analysis, where *p*-value < 0.05 is indicated as not normally distributed data. Descriptive analysis was conducted to present the median, quartile, mean, and standard deviation (SD). For normally distributed, data were shown in mean (SD); and for not normally distributed, data were shown in median (Q1–Q3). To evaluate the significant difference between groups, Mann–Whitney analysis was carried out. If the data were categorized, cross-tabulation was conducted with chi-square analysis. *P*-value < 0.05 indicates that there was a significant difference. All analyses were conducted using SPSS v. 23.0.

## Result

We recruited 474 participants who passed the screening stage, where the inclusion criteria were fulfilled. At the end of the data collection process, there were 382 participants who finished the process. After excluding participants who did not comply with the eligibility criteria, analyses incorporated 380 participants where 82.4% of them were students. Table 1 shows the characteristics of participants based on occupation status. It is shown that this study is dominated by women, who contribute up to 73.4% of the total participants. More than half of the participants are unmarried (88.2%) and from health science faculty (62.9%). In nutritional status, we found that there are participants who are underweight (52 participants, 13.7%) and obese (92 participants, 24.2%), beside normal (177 participants, 46.6%) and overweight (59 participants, 15.5%).

**TABLE 1:** Subject characteristics (*n* = 380).

	Students *n* (%)	Employee *n* (%)	Total *n* (%)
**Sex**			
Woman	231 (73.8)	48 (71.6)	279 (73.4)
Man	82 (26.2)	19 (28.4)	101 (26.6)
**Residence**			
Java	198 (63.3)	48 (71.6)	246 (64.7)
Other than Java	115 (36.7)	19 (28.4)	134 (35.3)
Age [Median (Q1–Q3)]	22 (20–23)	28 (26–31)	22 (20–25)
Youth (≤24)	269 (85.9)	9 (13.4)	278 (73.2)
Non-youth (>24)	44 (14.1)	58 (86.6)	102 (26.8)
**Marital status**			
Not Married	298 (95.2)	37 (55.2)	335 (88.2)
Married	15 (4.8)	30 (44.8)	45 (11.8)
**Education Field**			
Health science	194 (62.0)	45 (67.2)	239 (62.9)
Technology science	40 (12.8)	5 (7.5)	45 (11.8)
Social science	34 (10.9)	2 (3.0)	36 (9.5)
Other	45 (14.4)	15 (22.4)	60 (15.8)
**University**			
USU	45 (14.4)	11 (16.4)	56 (14.7)
UNILA	59 (18.8)	5 (7.5)	64 (16.8)
UI	24 (7.7)	39 (58.2)	63 (16.6)
UNIMUS	75 (24.0)	8 (11.9)	83 (21.8)
UNS	110 (35.1)	4 (6.0)	114 (30.0)
Nutritional status [Median (Q1–Q3)]	21.8 (19.6–24.4)	23.3 (21.5–26.9)	22.1 (19.8–25.0)
Underweight	49 (15.6)	3 (4.5)	52 (13.7)
Normal	150 (47.9)	27 (40.3)	177 (46.6)
Overweight	44 (14.1)	15 (22.4)	59 (15.5)
Obese	70 (22.4)	22 (32.8)	92 (24.2)
Total	313 (82.4)	67 (17.6)	380 (100.0)

USU, Universitas Sumatera Utara; UNILA, Universitas Lampung; UI, Universitas Indonesia; UNIMUS, Universitas Muhammadiyah Semarang; UNS, Universitas Sebelas Maret.

Table 2 shows the difference intake based on sex, residencies, age, marital status, education background, university, and nutritional status. Overall participants present the total fluid intake as much as 1,670 (1,326–2,034) ml/day with water as the largest contributor and followed by SSBs. Based on sex, it appears that male participants consume more daily total fluid and water compared to female participants (1,832 vs. 1,612 ml/day; *p*-value = 0.002 and 1,388 vs. 1,239 ml/day; *p*-value = 0.029). Based on residencies, participants who reside in Java present a lower consumption of water (1,216 vs. 1,412 ml/day; *p*-value = 0.002) but higher in SSBs (214 vs. 162 ml/day; *p*-value = 0.018). Compared to participants who are fairly active, participants who show a low physical activity display a lower total fluid intake (1,628 vs. 1,768 ml/day; *p*-value = 0.025). In contrast, overweight participants show a higher intake of total fluid intake (1,578 vs. 1,832 ml/day; *p*-value = 0.000) and water intake (1,183 vs. 1,451 ml/day; *p*-value = 0.000) compared to non-overweight participants. We did not find any significant difference between participants based on educational background and knowledge.

**TABLE 2:** Fluid intake water and beverages according to subjects’ characteristics of different groups (ml/day).

Characteristic	Total	Water	Hot beverages	Milk and derivatives	SSB	Juices	Other
Total subjects	1,670 (1,326–2,034)	1,262 (983–1,666)	0 (0–0)	0 (0–40)	200 (91–350)	0 (0–0)	30 (0–80)
**Education background**							
Health science	1,664 (1,314–2,018)	1,248 (975–1,667)	0 (0–0)	0 (0–37)	186 (75–363)	0 (0–0)	0 (0–78)
Non-health science	1,693 (1,354–2,091)	1,305 (1,004–1,659)	0 (0–0)	0 (0–50)	211 (106–306)	0 (0–0)	43 (0–81)
**Sex**							
Male	1,832 (1,495–2,183)	1,388 (1,113–1,760)	0 (0–0)	0 (0–55)	211 (103–364)	0 (0–0)	40 (0–86)
Female	1,612 (1,291–1,963)*	1,239 (948–1,630)*	0 (0–0)	0 (0–34)	194 (75–348)	0 (0–0)	29 (0–75)
**Age**							
≤24 years old	1,662 (1,307–2,033)	1,262 (981–1,669)	0 (0–0)	0 (0–34)	184 (85–341)	0 (0–0)	28 (0–75)
>24 years old	1,692 (1,370–2,063)	1,276 (990–1,665)	0 (0–27)*	0 (0–47)	229 (111–376)	0 (0–0)	37 (0–83)
**Area**							
Java	1,635 (1,314–1,983)	1,216 (935–1,630)	0 (0–0)	0 (0–50)	214 (93–376)	0 (0–0)	37 (0–83)
Outside Java	1,770 (1,357–2,100)	1,412 (1,125–1,750)*	0 (0–0)*	0 (0–29)*	162 (79–315)*	0 (0–0)	0 (0–75)
**Occupation**							
Employee	1,710 (1,360–2,231)	1,293 (993–1,700)	0 (0–24)	0 (0–41)	232 (97–349)	0 (0–0)	36 (0–83)
Student	1,661 (1,312–2,019)	1,262 (982–1,661)	0 (0–0)*	0 (0–40)	198 (85–350)	0 (0–0)	29 (0–79)
**Marital status**							
Not married	1,693 (1,384–2,054)	1,249 (889–1,553)	0 (0–51)	0 (0–43)	250 (145–422)	0 (0–0)	57 (0–83)
Married	1,664 (1,313–2,035)	1,263 (993–1,681)	0 (0–0)*	0 (0–38)	188 (84–343)*	0 (0–0)	22 (0–75)*
**Physical activity**							
Low	1,628 (1,305–1,935)	1,239 (949–1,625)	0 (0–0)	0 (0–43)	209 (93–352)	0 (0–0)	30 (0–78)
Fair	1,768 (1,345–2,156)*	1,339 (991–1,743)	0 (0–0)	0 (0–38)	187 (85–343)	0 (0–0)	30 (0–83)
**Knowledge**							
Low	1,715 (1,333–2,070)	1,281 (1,018–1,625)	0 (0–0)	0 (0–43)	191 (73–368)	0 (0–0)	34 (0–75)
Fair	1,647 (1,321–2,020)	1,239 (927–1,702)	0 (0–0)	0 (0–37)	202 (99–339)	0 (0–0)	28 (0–82)
**Nutritional status**							
Underweight	1,384 (1,123–1,872)^†^	1,116 (801–1,460)	0 (0–0)	0 (0–50)	144 (45–323)	0 (0–0)	41 (0–89)
Normal	1,632 (1,290–1,954)	1,214 (903–1,631)	0 (0–0)	0 (0–66)	201 (94–358)	0 (0–0)	36 (0–82)
Overweight	1,643 (1,350–2,100)	1,319 (1,059–1,756)	0 (0–0)	0 (0–26)^†^	177 (75–328)	0 (0–0)	29 (0–73)
Obese	1,917 (1,608–2,199)^†^	1,530 (1,203–1,866)^†^	0 (0–0)	0 (0–28)^†^	239 (101–361)	0 (0–0)	0 (0–75)

Data are presented in median (Q1–Q3).

SSB, sugar-sweetened beverages.

Significance of difference was analyzed using Mann–Whitney test.

*Indicates significant difference between groups.

^†^Indicates significant difference compared to the normal group.

P-value was set to 0.05 to indicate significant difference.

Results on water and types of beverages intake based on drinking occasion can be found in Table 3. There are three drinking occasions, namely, iftar or breakfasting, nighttime, and suhoor or pre-dawn meal. The notable difference is seen in total fluid, water, and SSB intake. Among the three drinking occasions, participants consume total fluid (560 vs. 474 vs. 574 ml/day) and water (489 vs. 279 vs. 483 ml/day) the least at iftar significantly. In contrast, SSB is consumed the most at iftar (0 vs. 101 vs. 43 ml/day), compared to two other drinking occasions.

**TABLE 3:** Water and types of beverages intake based on drinking time (*n* = 380).

Beverages type	Iftar	Night time	Suhoor	Total
Total	474 (375–590)^a^	574 (414–810)^b^	560 (423–711)^c^	1,670 (1,326–2,034)
Water	279 (146–426)^a^	483 (32–687)^b^	489 (367–650)^b^	1,262 (983–1,666)
Hot Beverages	0 (0–0)^a^	0 (0–0)^b^	0 (0–0)^b^	0 (0–0)
Milk and derivatives	0 (0–0)^a^	0 (0–0)^b^	0 (0–0)^c^	0 (0–40)
SSB	101 (22–213)^a^	43 (0–100)^b^	0 (0–60)^c^	200 (91–350)
Juices	0 (0–0)^a^	0 (0–0)^b^	0 (0–0)^c^	0 (0–0)
Other	0 (0–63)^a^	0 (0–0)^b^	0 (0–0)^c^	30 (0–80)

SSB, sugar-sweetened beverages.

Difference in uppercase alphabets indicates significantly different amounts within types of beverages. Data are presented in median (Q1–Q3). Different uppercase letters indicate a significant difference between occasions. The significance of difference was analyzed using Mann–Whitney test. P-value was set to 0.05 to indicate significant difference.

Table 4 shows the followed pattern of water consumption during Ramadan. Most participants (196 participants, 51.6%) practice the 2-4-2 pattern that indicates two glasses at iftar, four glasses at nighttime, and two glasses at suhoor. There are still 74 participants (19.5%) who adopt 2-2-2 pattern that accumulates only six glasses per day and 30 participants (7.9%) who do not adopt any particular pattern. Based on the pattern, 4-2-2 pattern shows a significant difference (*p*-value = 0.006; OR = 2.774) to achieve adequate intake compared to participants who use other pattern. In contrast, the practice of drinking eight glasses of water daily, regardless of the pattern, shows significant difference to achieve adequate intake compared to participants who do not adopt the eight glasses of water daily pattern (*p*-value = 0.020; OR = 1.871).

**TABLE 4:** Pattern of drinking habit and its association with adequate water intake (*n* = 380).

Drinking habit	Inadequate *n* (%)	Adequate *n* (%)	*P*-value	OR (Confidence interval)
Not Drinking 2-2-2	198 (64.7)	108 (35.3)	0.743	0.880
Drinking 2-2-2	50 (67.6)	24 (32.4)		(0.513–1.510)
Not drinking 2-2-4	219 (65.4)	116 (34.6)	1.000	1.042
Drinking 2-2-4	29 (64.4)	16 (35.6)		(0.543–1.996)
Not DRINKING 2-4-2	122 (66.3)	62 (33.7)	0.760	1.093
Drinking 2-4-2	126 (64.3)	70 (35.7)		(0.716–1.669)
Not drinking 4-2-2	233 (67.5)	112 (32.5)	0.006*	2.774
Drinking 4-2-2	15 (42.9)	20 (57.1)		(1.396–5.621)
Not drinking 8 glasses	78 (75.0)	26 (25.0)	0.020*	1.871
Drinking 8 glasses in total	170 (61.6)	106 (38.4)		(1.128–3.102)

Drinking habit number indicated in the order iftar—nighttime—suhoor.

Significance of difference was analyzed using cross-tabulation analyses. P-value was set to 0.05 to indicate significant difference. *Indicates significant difference between groups.

## Discussion

The result of this study has indicated that adults did not consume adequate water during Ramadan; their total fluid intake was lower than the recommendation [1,670 (1,326–2,034) ml/day]. Leiper et al. presented that due to restricted fluid intake during Ramadan, Ramadan fasting can lead up to a few health issues, for example, increase in irritability along with physical weariness (26). Then, it is important to have a drinking pattern to achieve daily recommendation. In this study, the most widely used pattern to adhere to water recommendations was drinking in a pattern of 2-4-2 glasses in the order of iftar—nighttime—suhoor (196 participants, 51.5%). In contrast, the total fluid intake of participants with a drinking pattern of 4-2-2 was significantly higher [*p*-value = 0.006; OR (confidence interval): 2.774 (1.396–5.621)]. This result showed that participants who had a drinking pattern of 4-2-2 had a better chance of achieving daily water recommendation.

The Indonesian Ministry of Health has released the recommended daily water intake in Indonesia RDA (2019), which stated that the daily water intake of men and women is 2,500 and 2,350 ml/day, respectively (27). The amount includes water from food, which can be varied according to food culture in the country, age, and sex (22). In this study, we referred to Laksmi et al., where the research was also conducted in Indonesia (8), and the total daily fluid recommendation was reduced by 20% since this study did not include water from food during the assessment. Despite the limited time available for people to consume water and beverages during Ramadan, it is necessary to achieve the daily intake recommendation to optimize the performance of daily activity (26). This is the first research conducted in Indonesia by using fluid diary through an online questionnaire during Ramadan in order to investigate fluid intake among adults.

Ibrahim et al. found a higher intake of daily fluid during Ramadan among Indonesian adults, which was 2,305 ml/day (28). This number might include water from food since the food record method was utilized in collecting the data on the fluid intake. Among female students in Iran, water and beverage intake was slightly lower (1,512 ± 620 ml/day) than in our study (29). Many studies were done on athletes to investigate the water intake alteration effect on athletes’ performance during Ramadan. In Tunisia, three studies were conducted: one on physically active men, one on bodybuilders, and one on rugby athletes, and the result of the daily water intake was 3,300, 3,800, and 3,400 ml/day (30–32). Significantly higher physical activity undoubtedly was responsible for the higher water intake among athletes (33).

In the meantime, the differences in water and beverage intake based on educational background and knowledge level did not show any significance in spite of a higher level of hydration knowledge among health science participants, which have failed to meet the water intake recommendation. Beyond knowledge, health practice, specifically healthy hydration practice, was determined by various factors; for instance, water availability, acceptability, affordability, accessibility, safety, and sufficiency (34). The notable difference was found in participants who were female, lived outside Java, or were not overweight who presented lower water intake significantly compared to their counterparts. Among countries in Latin America, China, and Indonesia, water intake discrepancy between male and female participants was inconclusive (8, 35, 36). Even though this study matched the expectation, that women had a lower water intake recommendation due to their lower body mass and body water percentage compared to men, both parties did not achieve the recommendation (13). Kim and Yang showed this resemblance where higher lean mass and BMI led to higher water intake among adult men and women, but higher fat mass did not (37).

In terms of SSB intake, participants who lived outside Java or were married showed a significantly lower intake compared to their counterparts. The different geographical area between Java and non-Java area might contribute to the water and SSB intake. Java Island, especially Jakarta and its sub-urban area, is considered to be more developed compared to other islands since the capital city is located on Java Island (38). This condition resulted in a gap in economy, education, population distribution, and infrastructure that led to food accessibility gap between the two areas. The earlier evidence proved that the background of different intake between residencies might be due to the limited access to certain food that could drive consumers to decrease their food consumption (39, 40). Along with a higher intake of SSBs, unmarried participants also showed a higher intake of other beverages compared to the married participants. Compared to Laksmi et al., this study showed a higher intake of other beverages among Indonesian adults because of the Ramadan tradition, where usually they prepared sweet beverages for energy replenishment. Shatila et al. study during Ramadan showed that the energy contribution from SSBs significantly increased compared to before Ramadan, which was 4.7 ± 3.2% to 8.4 ± 10.1% (41).

In comparison, three occasions of drinking time were considered, iftar or breakfasting, nighttime, and suhoor or pre-dawn meal. Among these, iftar is where water and beverages were consumed the least and nighttime was the most (iftar: 474 ml/day; nighttime: 574 ml/day; suhoor: 560 ml/day). Considering that nighttime is longer compared to suhoor and iftar, it was only natural for participants to consume water and beverages the most during the nighttime. Another issue that needs to be highlighted is the fact that the consumption of SSBs as well as other beverages during iftar was significantly higher than that during suhoor and nighttime. Even though the increase in sugar-containing food during Ramadan was similar to previous studies, the drinking occasion was not explored (15, 41, 42). The results resembled what Chia et al. found on the assessment change of restricted diet during Ramadan fasting that Ramadan fasting led to higher temptation to consume unhealthy food (43). However, in spite of this, studies did not find eating behavior disordered due to Ramadan fasting among adolescence (44, 45).

As consumption time during Ramadan is limited, the participants were asked about their drinking habits to meet the recommendation before fluid recording started. The most adapted drinking pattern in this study was the 2-4-2 pattern in the order of iftar—nighttime—suhoor (196 participants, 51.6%). But actually the least-followed drinking pattern 4-2-2 in the order of iftar—nighttime—suhoor showed a significant association with water intake adequacy [*p*-value = 0.006; OR (CI) = 2.774 (1.396–5.621)]. Drinking eight glasses per day, regardless of the pattern, was significantly associated with adequate water intake compared to those who only drink six glasses of water daily with the pattern of 2-2-2 [*p*-value = 0.020; OR (CI) = 1.871 (1.128–3.102)]. This result might bring awareness to the community to drink eight glasses of water on a daily basis with a pattern of 4-2-2 glasses in order to achieve adequate water intake. Achieving the adequate daily water intake is very important during Ramadan fasting since Ramadan fasting is 30 days long and dehydration may weaken immunity, which can cause a higher risk of morbidity (46).

The approach on how to fulfill daily fluid recommendation is needed since people tend to consume a lesser amount of water during Ramadan (12, 18). Several studies showed the approach and management to maintain water intake during Ramadan for people with illnesses, especially people with diabetes (47–49). Hamdy et al., in diabetes alliances Ramadan guideline, encouraged patients with diabetes to consume 40–50% calorie intake during iftar, 30–40% during suhoor, and the rest for snack between meals (50). However, we did not find a plan or guideline on water intake for healthy adults during Ramadan fasting, admitting that dehydration is not favored during the daily activities. Abinowo reported that female adults in Indonesia consumed water mostly during iftar and suhoor, but did not suggest any hydration plan to achieve water recommendation (51).

There were a few limitations in this study that needed to be considered. Although this study did not assess the fluid intake contained in food, we investigated the fluid intake from beverages in the most extensive way possible. We also conducted this study using an online questionnaire to follow the COVID-19 health protocol, which may result differently compared to offline interviews. Trained enumerators were hired to overcome this constraint and ensure the participants filled the questionnaire accordingly. The most relevant strength of this study was that we utilized Liq.In7, which has been validated to assess fluid intake from beverages by Johnson et al. (19).

In conclusion, this study, investigating fluid intake and its pattern among Indonesian adults during Ramadan, showed that the total fluid intake was below the recommendation and water consumption among adults in Indonesia needs to be improved. Water contributed the most to total daily intake, followed by SSBs and other beverages. Based on the drinking occasion, participants’ consumption was the highest during nighttime and then subsequently during suhoor and iftar. The most commonly used drinking pattern was 2-4-2, but a drinking pattern of 4-2-2 for iftar—nighttime—suhoor order may be adapted during Ramadan as an approach to achieve daily water recommendation, although eight glasses of water a day in total was already effective.

## Data availability statement

The original contributions presented in this study are included in the article/supplementary material, further inquiries can be directed to the corresponding author.

## Ethics statement

The studies involving human participants were reviewed and approved by Prof. Dr. Rita Sita Sitorus, Ph.D., Sp.M(K). The patients/participants provided their written informed consent to participate in this study.

## Author contributions

DS, DC, BM, and DF designed the study and investigated the data collection. DS, DC, BM, NM, WL, and PI analyzed the initial data and manuscript writing. AA, DM, FS, DKS, and YU investigated the data collection and manuscript writing. All authors were involved in writing process of the manuscript and gave final approval upon the submitted versions.
